# Synaptosomal Mitochondrial Dysfunction in 5xFAD Mouse Model of Alzheimer's Disease

**DOI:** 10.1371/journal.pone.0150441

**Published:** 2016-03-04

**Authors:** Lu Wang, Lan Guo, Lin Lu, Huili Sun, Muming Shao, Simon J. Beck, Lin Li, Janani Ramachandran, Yifeng Du, Heng Du

**Affiliations:** 1 Department of Biological Sciences, The University of Texas at Dallas, Richardson, TX, United States of America, 75080; 2 Shandong University, Shandong Provincial Hospital, Jinan, Shandong Province, China, 250100; 3 Shenzhen Traditional Medicine Hospital, Shenzhen, Guangdong Province, China, 518031; Nathan Kline Institute and New York University School of Medicine, UNITED STATES

## Abstract

Brain mitochondrial dysfunction is hallmark pathology of Alzheimer’s disease (AD). Recently, the role of synaptosomal mitochondrial dysfunction in the development of synaptic injury in AD has received increasing attention. Synaptosomal mitochondria are a subgroup of neuronal mitochondria specifically locating at synapses. They play an essential role in fueling synaptic functions by providing energy on the site; and their defects may lead to synaptic failure, which is an early and pronounced pathology in AD. In our previous studies we have determined early synaptosomal mitochondrial dysfunction in an AD animal model (J20 line) overexpressing human Amyloid beta (Aβ), the key mediator of AD. In view of the limitations of J20 line mice in representing the full aspects of amyloidopathy in AD cases, we employed 5xFAD mice which are thought to be a desirable paradigm of amyloidopathy as seen in AD subjects. In addition, we have also examined the status of synaptosomal mitochondrial dynamics as well as Parkin-mediated mitophagy which have not been previously investigated in this mouse model. In comparison to nontransgenic (nonTg mice), 5xFAD mice demonstrated prominent synaptosomal mitochondrial dysfunction. Moreover, synaptosomal mitochondria from the AD mouse model displayed imbalanced mitochondrial dynamics towards fission along with activated Parkin and LC3BII recruitment correlating to spatial learning & memory impairments in 5xFAD mice in an age-dependent manner. These results suggest that synaptosomal mitochondrial deficits are primary pathology in Aβ-rich environments and further confirm the relevance of synaptosomal mitochondrial deficits to the development of AD.

## Introduction

Mitochondria play a crucial role in sustaining neuronal function and survival. Accordingly, mitochondrial impairments have been linked to the pathogenesis of a number of neurodegenerative diseases including Alzheimer's disease (AD) [1, 2]. Previous studies have repeatedly reported that in AD cases and in AD animal models brain mitochondria undergo severe injuries including impaired oxidative phosphorylation (OXPHOS), dampened mitochondrial membrane potential, and increase production of reactive oxygen species (ROS) as well as enhanced mitochondrial permeability transition pore (mPTP) formation [3–9]; and these neuronal mitochondrial abnormalities are closely associated with the toxicity of the key AD mediator, Amyloid beta (Aβ) [3, 4, 7, 10–12]. Notably, a subgroup of neuronal mitochondria also known as synaptosomal mitochondria which specifically reside at synapses play a critical role in providing energy and regulating calcium homeostasis locally to maintain synaptic function and support synaptic plasticity [13]. Different from their siblings in the soma, their sparse distribution and constant activation confer susceptibility to synaptosomal mitochondria to accumulate damages leading to mitochondrial abnormalities in physiological and pathological conditions [13–15]. Given the crucial role of synaptosomal mitochondria in supporting synaptic function and synaptic failure as an early cardinal sign of AD, it is of paramount importance to understand synaptosomal mitochondrial defects in Aβ-rich conditions.

In our previous studies by using an AD mouse model J20 line expressing the human APP695/751/770 with mutations at K670N/M671L and V717F, we have found the early defects in synaptosomal mitochondria which exaggerates with age [3, 6] suggesting that synaptosomal mitochondrial dysfunction is a pronounced brain pathological change in response to Aβ toxicity in this mouse model. However, it has been suggested that J20 transgenic model has its limitations in representing the full aspects of amyloidopathy in AD cases [16]; and it would be desirable to examine synaptosomal mitochondrial dysfunction in different animal models with a better representation of amyloidopathy in AD brains. In this study, we employed 5xFAD mice which express the human APP with the mutations at K670N/M671L and I716V/V717I as well as human presenilin 1 (PS1) mutations at M146V and L286V [17]. In comparison to J20 line, 5xFAD mice demonstrate a faster deposition of Aβ plaques, higher levels of intra-neuronal Aβ accumulation, neuro-inflammation as well as neuronal loss which make 5xFAD mice a desirable model mimicking amyloidopathy in AD cases [17–19]. Notably, synaptosomal mitochondrial function has never been investigated in 5xFAD mice. In addition, the status of mitochondrial dynamics as well as the activation of Parkin-mediated mitophagy in synaptosomal mitochondria has not been comprehensively investigated in this AD mouse model. The examination of synaptosomal mitochondrial dysfunction in 5xFAD mice will further strengthen our hypothesis that Aβ toxicity confers susceptibility to synaptosomal mitochondria in the development of AD.

## Materials and Methods

### Mice

Animal studies were approved by the University of Texas at Dallas Institutional Animal Care and Use Committee (IACUC) and in accordance with the National Institutes of Health guidelines for animal care. 5xFAD mice overexpress a human form of APP-bearing mutations (SwFlLon) and PSEN1 mutations (M146L and L286V) linked to familial AD. 5xFAD mice were obtained from Jackson Laboratory. The genotype of all the transgenic animals were doubled checked by performing PCR before the experiments. Any mouse with wrong genotyping was excluded and euthanized by CO2 inhalation. Mice were housed (4 animals per cage) with a 12/12 hr light/dark cycle and with *ad libitum* access to food and water. Animals were sacrificed by cervical dislocation for the brain mitochondrial purification. The number of mice was determined by our previous data and power calculation. Statistical Analysis was performed to determine whether the combinations of different types of mice with varied ages are statistically different for experimental designs. If the analysis indicated it was valid to continue, individual group and the combinations of group × age were compared by use of the Bonferroni corrected t-test. Based on our design, we performed power calculation to see whether we have enough power to detect the differences (http://www.stat.uiowa.edu/~rlenth/Power/). However, based on our experience, effect sizes are sometimes larger and so the actual number of animals used often can be considerably lower.

### Mitochondrial preparation

Synaptosomal mitochondria were prepared as described previously [3]. Briefly, brain cortices were placed in an ice-cold isolation buffer [225 mM mannitol, 75 mM sucrose, 2 mM K2PO4, 0.1% BSA, 5 mM Hepes, 1 mM EGTA (pH 7.2)]. The tissue was homogenized with a Dounce homogenizer (Kontes Glass Co.). The resultant homogenate was centrifuged at 1,300 × g for 5 min, and the supernatant was layered on adiscontinuous gradient containing 15%, 23%, and 40% (vol/ vol) Percoll and centrifuged at 34,000 × g for 8 min. After centrifugation, band 2 (the interface between 15% and 23% containing synaptosomes) and band 3 (the interface between 23% and 40% containing nonsynaptosomal mitochondria) were removed from the density gradient. The fractions were then resuspended separately in isolation buffer containing 0.02% digitonin and incubated on ice for 10 min. The suspensions were then centrifuged at 16,500 × g for 15 min. The resulting loose pellets were washed for a second time by centrifugation at 8,000 × g for 10 min. Pellets were collected and resuspended in isolation buffer. A discontinuous Percoll density gradient centrifugation was performed as described above for a second time. Band 3 was obtained and resuspended in isolation buffer to centrifuge at 16,500 × g for 15 min. The resultant pellet was washed in isolation buffer at 8,000 × g for 10 min. The final synaptosomal or nonsynaptosomal mitochondrial pellet was resuspended in isolation buffer and stored on ice. Protein concentration was determined using the Bio-Rad DC protein assay (BioRad Laboratories).

### Aβ Measurement

Aβ levels in mitochondrial fractions and brain cortex were measured by using human Aβ1–40 and Aβ1–42 ELISA kits (Invitrogen) following the manufacturer’s instructions [3].

### Immunoblotting Analysis

Equal amount of total protein samples were separated by SDS/PAGE (12% Bis-Tris gel; Life Technologies), and then transferred to PVDF membrane (BioRad Laboratories). After blocking in TBST buffer (20 mM Tris-HCl, 150 mM sodium chloride, 0.1% Tween-20) containing 5% (wt/vol) nonfat dry milk (Santa Cruz) for 1 h at room temperature, the membrane was then incubated with primary antibodies overnight at 4°C. This was followed by incubation with the corresponding secondary antibody for 1h at room temperature. The following antibodies were used: mouse anti-Parkin antibody (1:500; Santa Cruz), rabbit anti-LC3B (1:1000; Cell Signaling Technology), rabbit anti- β-amyloid (1:6000; Cell signaling Technology), rabbit anti-TOM40 (1:1000; Santa Cruz), rabbit anti- MFN2 (1:6000; Cell signaling Technology), mouse anti-OPA1 (1:5000; BD Transduction Lab), mouse anti- DLP1 (1: 2000; BD Transduction Lab), and goat anti-mouse IgG HRP conjugated and goat anti-rabbit IgG HRP conjugated (1:6000; Life Technologies). Image J software (National Institutes of Health) was used to analyze the scanned blots and to quantify protein signal intensity.

### Immunofluorescent staining

Brain tissues were fixed in 4% paraformaldehyde and embedded in paraffin blocks. After deparaffinization and rehydration, brain slices were boiled in citric buffer (10mM Citric acid, pH6.0) for 30min for antigen retrieval. Afterwards, the brain slices were blocked in 5% goat serum, 0.2%Triton in PBS for 1 hour followed by the incubation of primary antibodies overnight at room temperature. The staining was visualized by the labeling of corresponding secondary antibodies. Neuronal nuclei were stained by NISSL (1:200; Life Technologies). Antibodies used in the experiments: mouse anti-β-amyloid (1:1000; Cell signaling Technology), rabbit anti-F1FO ATP synthase β subunit (1:100; Santa Cruz), Alexa Fluor 488 conjugated goat anti-rabbit IgG (1:500; Life Technologies) and Alexa Fluor 594 conjugated goat anti-mouse IgG (1:500; Life Technologies). Images were collected using a Nikon confocal microscope and three dimension reconstruction of multiple images was conducted using Nikon NIS Advanced Research software.

### Mitochondrial respiration assays

Mitochondrial respiration assays were performed following our previously published method [20]. Purified mitochondria were energized by glutamate (5mM) and malate (5mM) and subjected to respiration assays on a Clark electrode. Oxygen consumption was triggered by the addition of ADP. Mitochondrial respiratory control ratio was defined as the ratio of State III respiration/State IV respiration.

### Mitochondrial ATP synthesis assay

Aliquots of mitochondria were analyzed by using the ATP Luminescent assay kit (Abcam). Briefly mitochondria were placed in isolation buffer [225 mM mannitol, 75 23 mM sucrose, 2 mM K2PO4, 0.1% BSA, 5 mM HEPES, 1 mM EGTA (pH 7.2)]. Mitochondria were energized with 5 mM glutamate/malate and ATP synthesis was induced with the injection of 500 μM ADP. ATP determination was measured following the manufacturer’s instructions.

### Neuron culture and axonal mitochondrial length measurement

Hippocampal neurons were cultured as previously described [3]. Briefly, hippocampi were dissected from day1 pups in cold HBBS (without Ca2+ and Mg2+) followed by dissociation with 0.05% trypsin at 37°C for 15 min and trituration with a Pasteur pipette in Neurobasal A medium (Invitrogen). Neurons were plated onto poly-D-lysine (Sigma—Aldrich)–coated Permanox Plastic chamber slides (NUNC) and cultured in Neurobasal A medium containing 2% B27 and 0.5 mM L-glutamine. Half of the culture medium was replaced by fresh medium every 3 d. Primary neurons were infected by lentivirus expressing mitochondrial targeted DsRed at days *in vitro* (DIV 8 days). Neurons at DIV21-22 days were used in the experiments. Axonal processes were determined as we previously described [3]. A process two to three times longer than other processes stemming from the soma is considered to be an axon. Particles with strong labeling (compared with background) and clear edges confined in axons were considered to be mitochondria. The length of mitochondria in the axonal segments (30–150μm) was measured by using Nikon NIS Advanced Research software.

### Electromicroscopy

Tissues were fixed with 4% paraformaldehyde, 0.1% glutaraldehyde in 0.1 M Phosphate buffer at 4°C for 24 h. After fixation, ultrathin sections were prepared and subjected to electromicrographs collection. Neurons from hippocampal CA1 region and cortical layer IV-V were selected. Glial cells were excluded based on their chromatin patterns [3, 53].

#### Morris Water Maze

Morris water maze was conducted to access the mice spatial learning and reference memory according to previously described protocol [54]. Briefly, mice were trained to find a submerged escape platform in an open swimming arena. Repeated trials (n = 4) were performed each day for 7 days by starting the mice at different non-congruent start locations (NW, N, NE, E, SE) while keeping the platform at a single location (SW). Each trial lasted 60 seconds with an additional 30 seconds learning time where mice were allowed to remain on the platform. After 7 days learning, mice were subjected to a probe test in which the platform was removed. Mice were analyzed for number of times they passed previous learning time platform location (SW). Behavior data was analyzed using HVS Image 2015 software (HVS Image).

#### Statistical Analysis

Two-way ANOVA followed by Bonferroni post hoc analysis or Student T Test were used for repeated measure analysis on SPSS software. P < 0.05 was considered significant. All data were expressed as the mean ± SEM.

## Results

### Extensive Aβ accumulation in synaptosomal mitochondria from 5xFAD mice

Mitochondrial accumulation of Aβ is a pronounced neuronal mitochondrial pathology and is thought to impose deleterious impact on mitochondrial function [3, 5, 20, 21]. We previously found that synaptosomal mitochondria from J20 line demonstrated early and more extensive Aβ accumulation in comparison to their nonsynaptosomal counterpart [3]. To determine the patterns of mitochondrial Aβ accumulation in 5xFAD mice, we employed 5xFAD mice and their control littermates lacking human form Aβ at the ages of 4 months old (mild to moderate brain Aβ deposition) and 9 months old (heavy brain Aβ deposition). Synaptosomal and nonsynaptosomal mitochondria were purified from the cortex of 5xFAD mice and their littermate controls, and the purity of the mitochondrial fractions was ascertained by the enrichment of mitochondrial specific proteins including translocase of outer mitochondrial membrane 40 (TOM40, an outer mitochondrial membrane protein), cytochrome c oxidase subunit IV (COXIV, an inner mitochondrial membrane protein) and heat shock protein 60 (Hsp60, a mitochondrial matrix protein) as well as the absence of calnexin (a specific marker of endoplasmic reticulum), and synaptophysin (a specific marker for synaptic vesicles) (S1 Fig).

**Fig 1:**
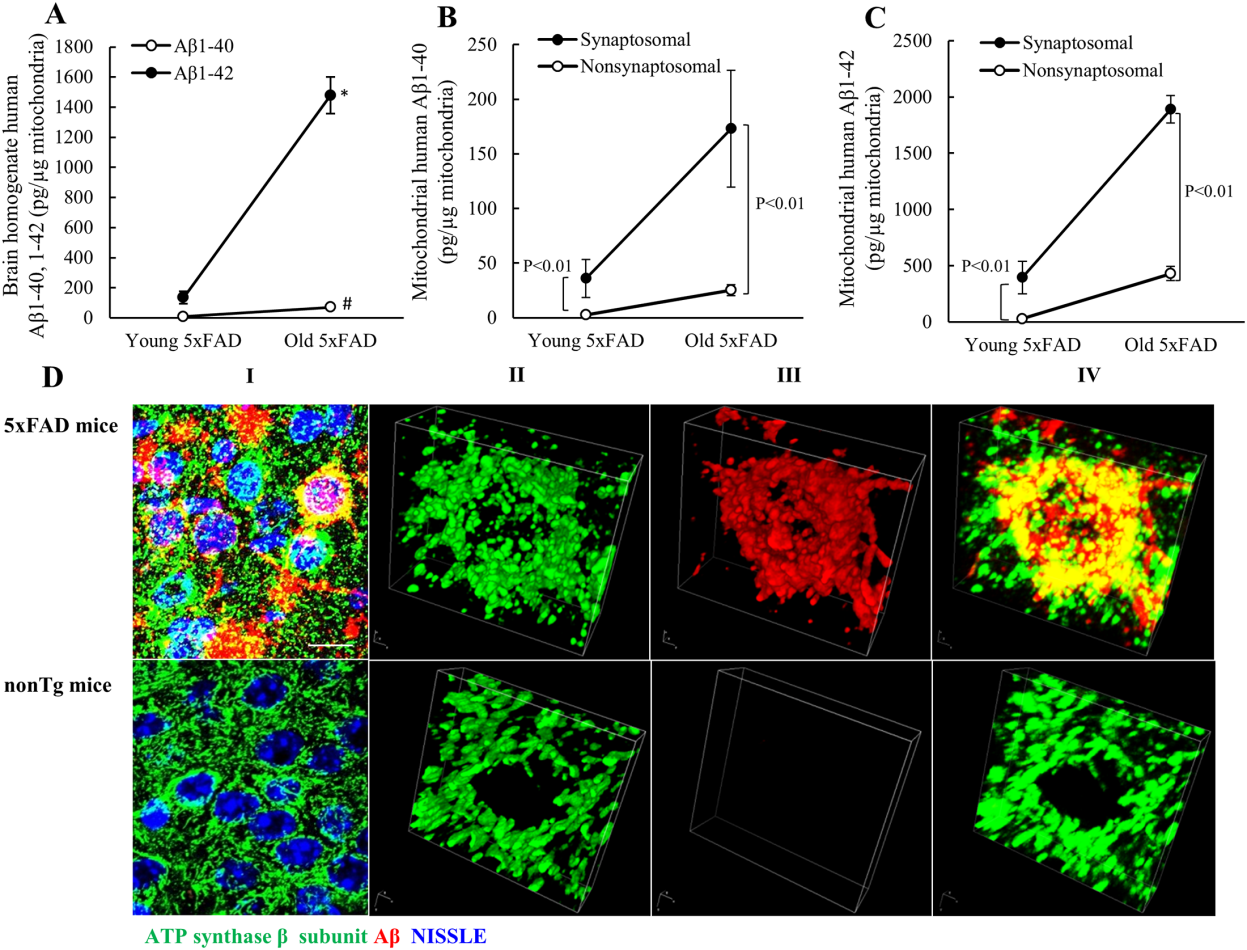
Aβ accumulation in synaptosomal mitochondria from 5xFAD mice at 4 (young) and 9 (old) months old. **(A)** Age-dependent brain Aβ1–40 and 1–42 deposition in 5xFAD mice. *, # P<0.05 vs the counterpart in young 5xFAD mice. **(B)** Age-dependent Aβ1–40 accumulation in synaptosomal and nonsynaptosomal mitochondria from 5xFAD mice. **(C)** Age-dependent Aβ1–42 accumulation in synaptosomal and nonsynaptosomal mitochondria from 5xFAD mice. N = 7 young mice and 8 old mice. **(D)** Aβ deposits in neurons, neuronal mitochondria and extracellular area in 5xFAD mice at 8 months old. Panels I shows the staining of Aβ (red), NISSL (blue) and F1FO ATP synthase β subunit (green). Panel II, III and IV represent three-dimension reconstructions of the F1FO ATP synthase β subunit staining, Aβ staining, and merged images, representatively. Scale bars = 20 nm.

Brain cortex and mitochondrial fractions from 5xFAD mice at 4 and 9 months old were subjected to ELISA assays for human form Aβ1–40 and 1–42, the two major Aβ species in AD. Brain cortex from 5xFAD mice exhibited an age-dependent increase in the levels of Aβ1–40 and 1–42 with significantly higher level of Aβ1–42 than that of Aβ1–40 at each tested age (Fig 1A). When we compared Aβ levels between synaptosomal and nonsynaptosomal mitochondria from 5xFAD mice, both mitochondrial fractions demonstrated an age-effect of Aβ1–40 (Fig 1B) and Aβ1–42 (Fig 1C) accumulation; whereas synaptosomal mitochondrial exhibited greater levels of Aβ1–40 (Fig 1B) and Aβ1–42 (Fig 1C) than their nonsynaptosomal counterpart at any given age. To ascertain the physical correlation of Aβ accumulation and neuronal mitochondria *in vivo*, we conducted confocal microscopy study using brain slices from nonTg and 5xFAD mice. Neurons were determined by the staining of NISSL and mitochondria were identified by the staining of mitochondrial F1FO ATP synthase β subunit. Neurons from 5xFAD mice demonstrated extensively overlapped staining of Aβ and the mitochondrial marker (Fig 1D, S2 Fig). Further three dimension reconstruction of multiple images showed that a significant part of intra-neuronal Aβ deposits in mitochondria (Fig 1D). These results conform to our previous findings in J20 mice [3]. In considering that synaptosomal mitochondria are specific from neurites while nonsynaptosomal mitochondria are a mixture of mitochondria from glial cells and neuronal soma [22], the results suggest that Aβ progressively deposits in both synaptosomal and nonsynaptosomal mitochondria from 5xFAD mice and neuron-specific synaptosomal mitochondria are more susceptible to Aβ accumulation.

### Impaired bioenergetics of synaptosomal mitochondria from 5xFAD mice

**Fig 2:**
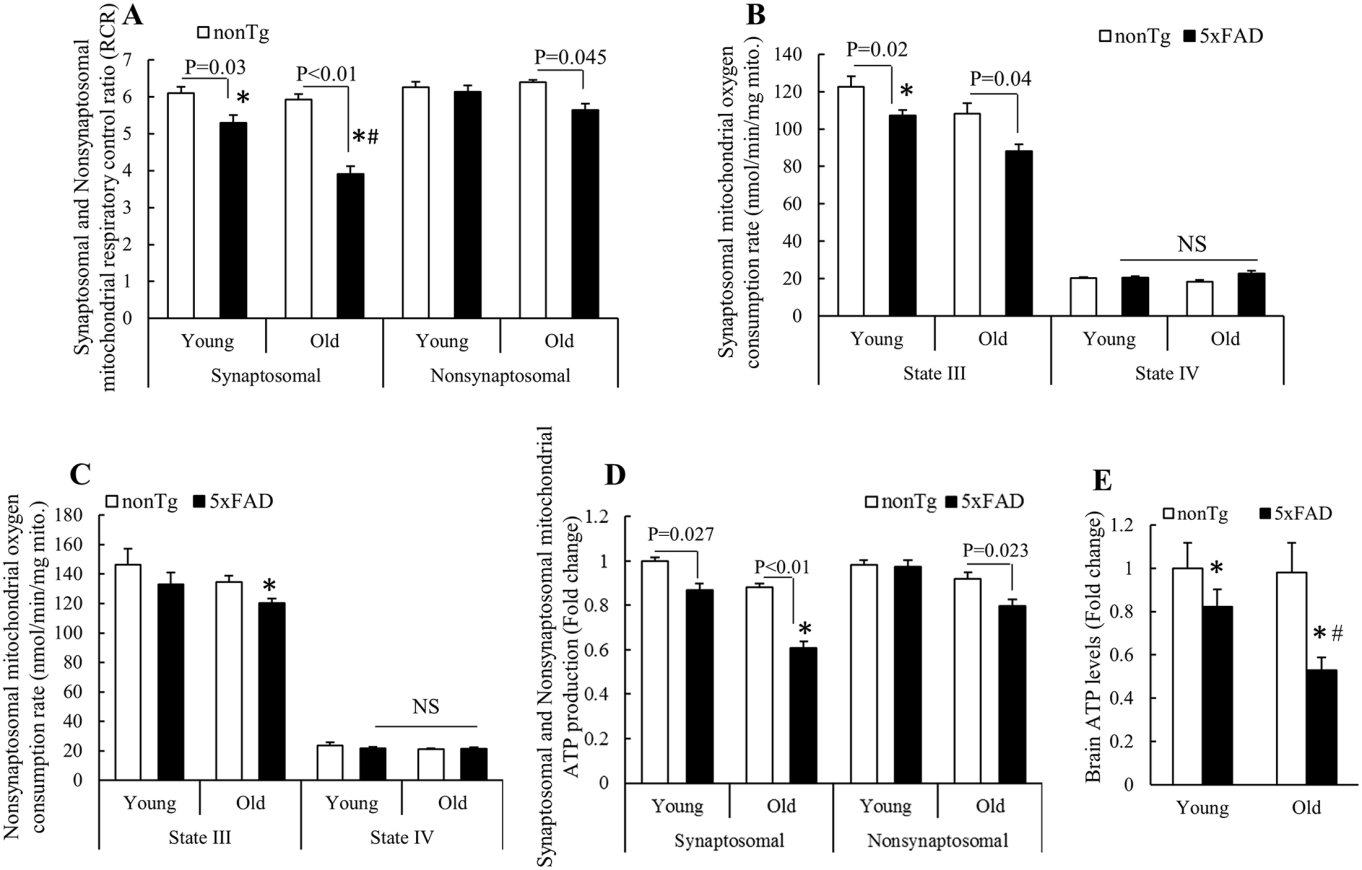
Impaired synaptosomal mitochondrial respiration in 5xFAD mice. **(A)** Mitochondrial RCRs of synaptosomal and nonsynaptosomal mitochondria from young and old 5xFAD and nonTg mice. *P<0.05 vs nonsynaptosomal counterpart. #P<0.05 vs other groups. **(B)** State III and Sate IV oxygen consumption rates of synaptosomal mitochondria from young and old nonTg and 5xFAD mice. *P<0.05 vs synaptosomal mitochondria from old 5xFAD mice. **(C)** State III and Sate IV oxygen consumption rates of nonsynaptosomal mitochondria from young and old nonTg and 5xFAD mice. *P<0.05 vs State III oxygen consumption rates of all the other groups. N = 3–4 mice of each group. **(D)** ATP production of synaptosomal and nonsynaptosomal mitochondria from young and old 5xFAD and nonTg mice. *P<0.05 vs all the other fractions. **(E)** Brain ATP levels in young and old nonTg and 5xFAD mice. *P<0.05 vs nonTg counterpart; #P<0.05 vs all the other groups. N = 6–7 mice of each group.

To determine whether Aβ accumulation leads to synaptosomal mitochondrial dysfunction, we examined mitochondrial bioenergetics, the abnormality of which is a well-documented mitochondrial defect in AD [1, 2]. To reflect mitochondrial bioenergetics, we first measured mitochondrial respiration on a Clark electrode. Synaptosomal and nonsynaptosomal mitochondria purified from 4 and 9 months old 5xFAD and nonTg mice were energized by glutamate and malate and the mitochondrial respiratory control ratio (RCR) was calculated as the ratio of State III oxygen consumption rate to State IV oxygen consumption rate. As shown in Fig 2A, at the mouse age of 4 months, synaptosomal mitochondria from 5xFAD mice demonstrated significantly lowered RCR (5.3 ± 0.21) in comparison to their counterparts from nonTg mice (6.1 ± 0.17) as well as nonsynaptosomal mitochondria from 5xFAD mice (6.1 ± 0.18) and nonTg mice (6.3 ± 0.14). Notably, there was no significant difference in the RCRs between nonsynaptosomal mitochondria from 5xFAD and nonTg mice at 4 months old indicating that nonsynaptosomal mitochondrial respiration were relatively preserved in this transgenic mouse model at young age (Fig 2A). When we measured the RCRs of mitochondrial fractions from 9 months old mice, synaptosomal mitochondria from 5xFAD mice exhibited an exaggerated decline in their RCR in comparison to other mitochondrial fractions (Fig 2A). Notably, both synaptosomal and nonsynaptosomal mitochondria from 5xFAD mice demonstrated an age-dependent reduction in their RCRs which correlates to the patterns of Aβ accumulation implicating the toxicity of Aβ on mitochondrial respiration.

We next compared State III and State IV respiration rate. As shown in Fig 2B & 2C, synaptosomal and nonsynaptosomal mitochondrial fractions from 5xFAD mice exhibited the changes in their State III oxygen consumption rates in correlation to the patterns of their RCRs; while there was no significant difference in State IV oxygen consumption rate between any mitochondrial fractions at the tested ages. The results suggest that mitochondria from different genotypes of mice at different ages were similarly preserved during preparation and lowered RCRs of mitochondria from 5xFAD mice are closely associated with their compromised ATP producing capacity.

Furthermore, we examined mitochondrial ATP producing capacity by measuring mitochondrial ATP synthesis. As shown in Fig 2D, synaptosomal mitochondria from young 5xFAD mice demonstrated significantly lowered ability in converting ADP to ATP in comparison to their counterparts from the age-matched nonTg mice; while the difference was enlarged in old mice. Similar to our findings of the changes of RCR, the preserved nonsynaptosomal mitochondrial ATP producing capacity in young 5xFAD mice was compromised with age (Fig 2D). Given mitochondria are the major source of ATP in brains, we then measured brain ATP levels. In agreement to impaired mitochondrial respiration and ATP producing capacity in 5xFAD mice, we have found significantly decreased brain ATP levels in 5xFAD mice in an age-dependent manner (Fig 2E).

### Deregulated synaptosomal mitochondrial dynamics in 5xFAD mice

**Fig 3:**
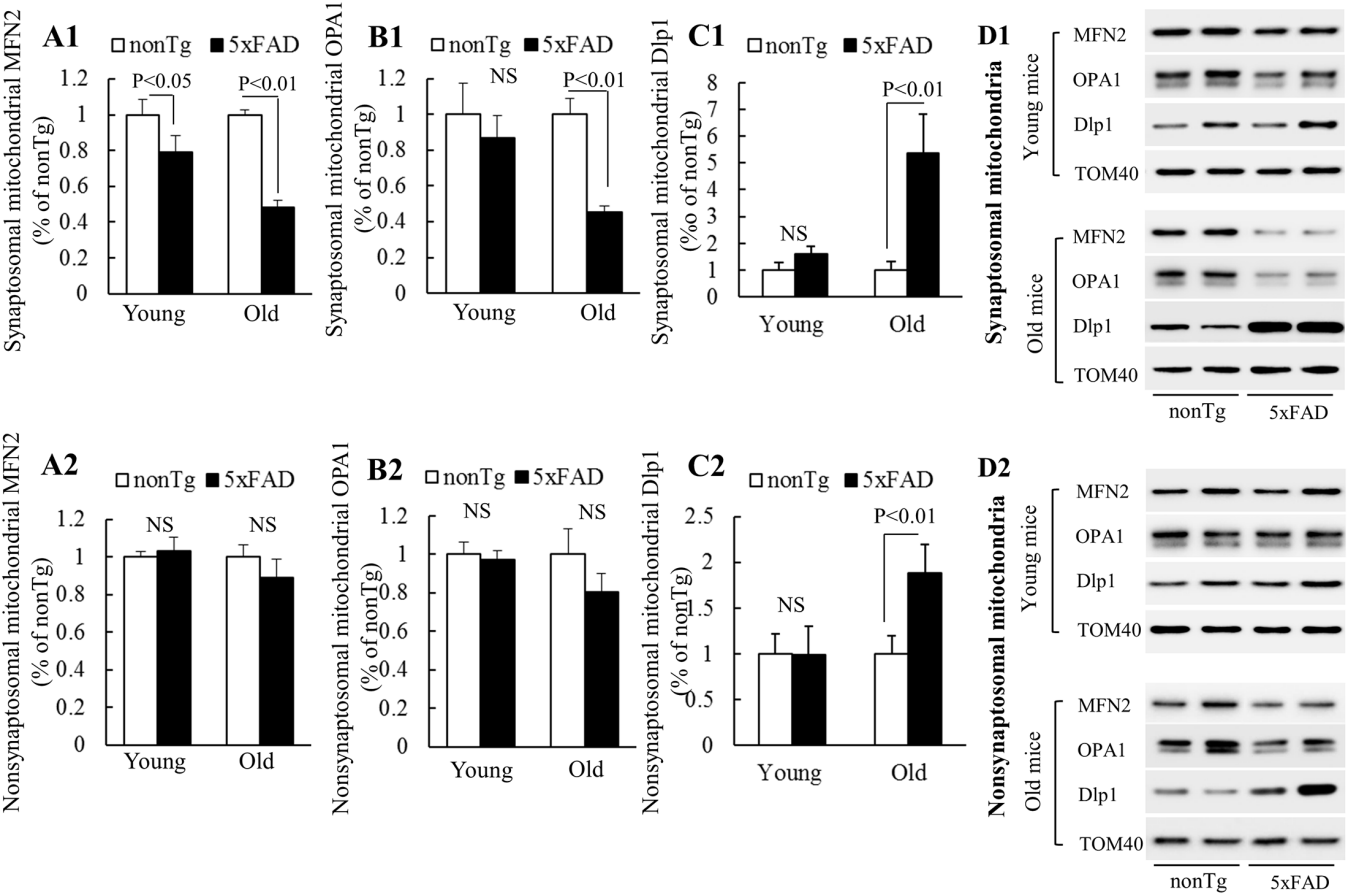
Altered mitochondrial fusion and fusion protein levels in synaptosomal mitochondria from 5xFAD mice. Synaptosomal and nonsynaptosomal mitochondria from 4 (Young) and 9 (Old) months old nonTg and 5xFAD mice were subjected to the immunoblots for the levels of MFN2 (**A1** synaptosomal mitochondria; **A2** nonsynaptosomal mitochondria), OPA1 (**B1** synaptosomal mitochondria; **B2** nonsynaptosomal mitochondria) and Dlp1 translocation (**C1** synaptosomal mitochondria; **C2** nonsynaptosomal mitochondria). **(D1)** and **(D2)** show representative immunoreactive bands from synaptosomal and nonsymaptosomal mitochondria, respectively. Mitochondrial TOM40 was used to determine the loading amount. N = 5–9 mice per group.

Mitochondria are highly dynamic organelles. Neuronal mitochondria constantly undergo fusion and fission to coordinate with their functional status as well as to adapt to neuronal energy demand [23]. Increased mitochondrial fission is also suggested to directly link to mitophagy via its role in segregation of damaged mitochondria [24]. To determine whether synaptosomal mitochondria from the AD mice display alterations in their dynamics, we examined the expression levels of mitochondrial fusion- and fission-related proteins in synaptosomal mitochondria. Synaptosomal and nonsynaptosomal mitochondria isolated from nonTg and 5xFAD mice were used for the immunoblotting assay to detect the expression levels of mitochondrial fusion proteins, mitofusin 2 (MFN2) and optic atrophy 1 (OPA1) and the recruitment of mitochondrial fission protein, dynamin-like protein 1 (Dlp1). Synaptosomal mitochondria from young 5xFAD mice exhibited significantly decreased MFN2 levels in comparison to other mitochondrial fractions (Fig 3A & 3D). In addition, we have also observed that synaptosomal mitochondria from young 5xFAD mice had mildly decreased OPA1 levels (Fig 3B & 3D) with slightly increased Dlp1 translocation (Fig 3C & 3D) in comparison to other mitochondrial fractions but with no statistical significance. However, synaptosomal mitochondria from old 5xFAD mice exhibited substantial reductions of MFN2 and OPA1 with a dramatic increase of Dlp1 translocation (Fig 3A–3D). Notably, nonsynaptosomal mitochondria from 5xFAD mice also displayed age-dependent changes of MFN2, OPA1 and Dlp1 but to a less significant degree in comparison to their synaptosomal counterpart. The suppression of mitochondrial MFN2 and OPA1 together with the elevation of mitochondrial Dlp1 translocation seems to suggest imbalanced synaptosomal mitochondrial dynamics towards fission in 5xFAD mice.

**Fig 4:**
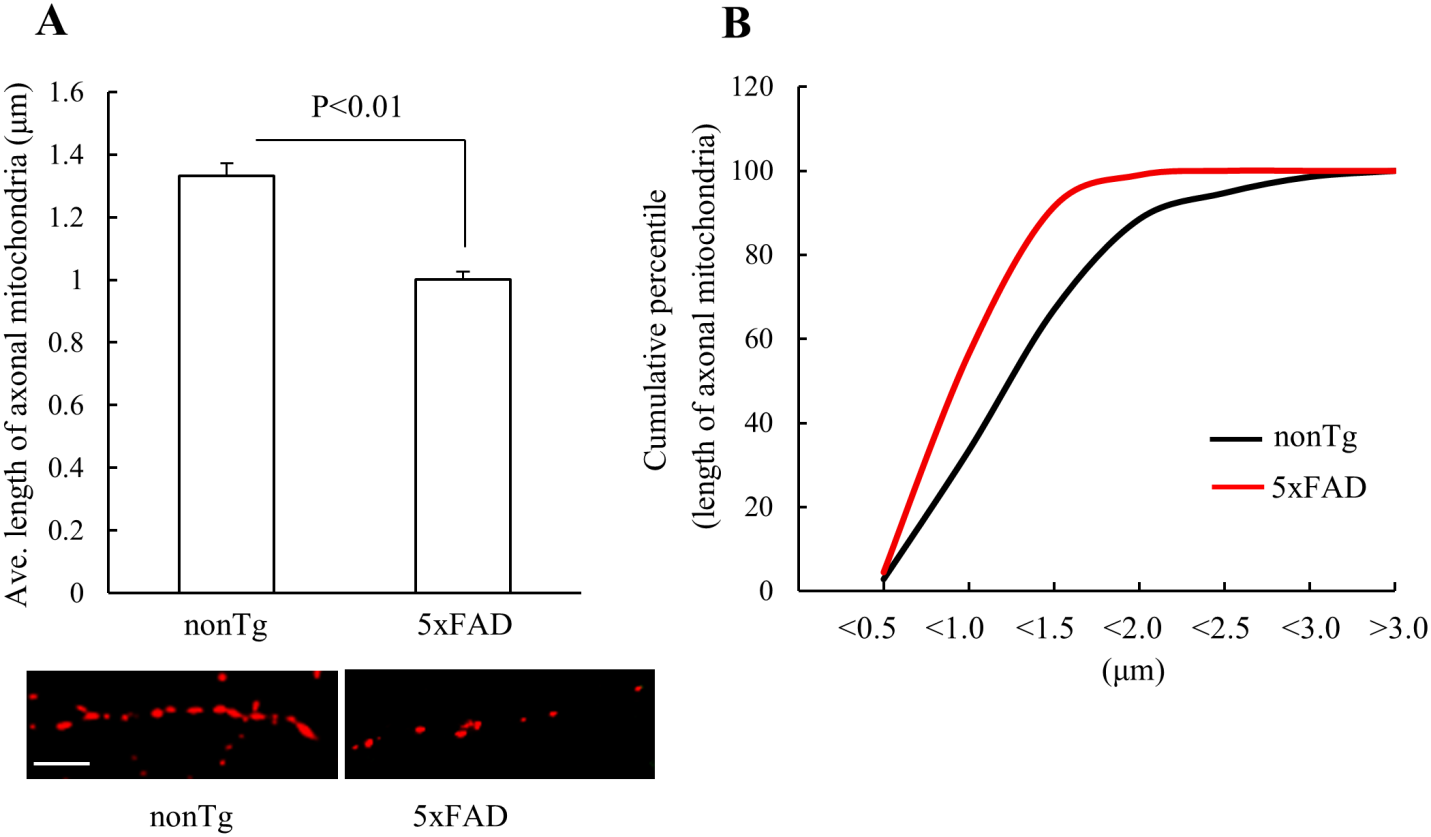
Decreased axonal mitochondrial length in cultured 5xFAD mouse neurons. **(A)** 5xFAD mouse neurons demonstrated significantly decreased axonal mitochondrial length in comparison to nonTg neurons. The lower panel are representative images. Scale bar = 5 μm. (**B**) Cumulative data for axonal mitochondrial length showing a leftward shift in 5xFAD mouse neurons. N = 8–12 neurons from each group.

If the above alterations of mitochondrial fusion and fission proteins could affect synaptosomal mitochondrial dynamics, 5xFAD mouse neuritic mitochondria would display elevated fragmentation resulting in decreased mitochondrial length. Due to the technical difficulty in accurately measuring neuritic mitochondrial length *in vivo*, we performed the experiments on primary cultured hippocampal neurons from nonTg and 5xFAD mice. Given the significance of axonal degeneration in AD [25], we chose to conduct quantitative analysis of the length of mitochondria in axonal segments (30–150 μm from soma). Our results showed that axonal mitochondria from 5xFAD mouse neurons demonstrated a significant decrease in their length in comparison to those from nonTg mouse neurons (Fig 4A & 4B) suggesting increased axonal mitochondrial fission in 5xFAD mouse neurons.

### Increased Parkin recruitment to synaptosomal mitochondria from 5xFAD mice

We have shown synaptosomal mitochondrial dysfunction in 5xFAD mice suggesting the quality control of synaptosomal mitochondria is severely impaired in the AD mouse model. Selected clearance of damaged mitochondria via macroautophagy, also known as mitophagy is a critical cellular mechanism to maintain a healthy pool of mitochondria [26–28]. Although the detailed mechanisms of mitophagy induction in neurons have not been fully defined, Parkin cascade has recently been identified to be the major pathway of mitophagy induction in neural cells [26, 28–31]. It's suggested that damaged neuritic mitochondria are tagged by Parkin and degraded via Parkin-mediated mitophagy in somatodendritic regions [26] or locally in the distal ends of neurites [31]. Therefore, we asked whether synaptosomal mitochondria from 5xFAD mice would undergo increased Parkin translocation given their severe injury. However, to the best of our knowledge, the information of the status of Parkin recruitment to synaptosomal mitochondria in AD mouse models *in vivo* is still extremely limited.

**Fig 5:**
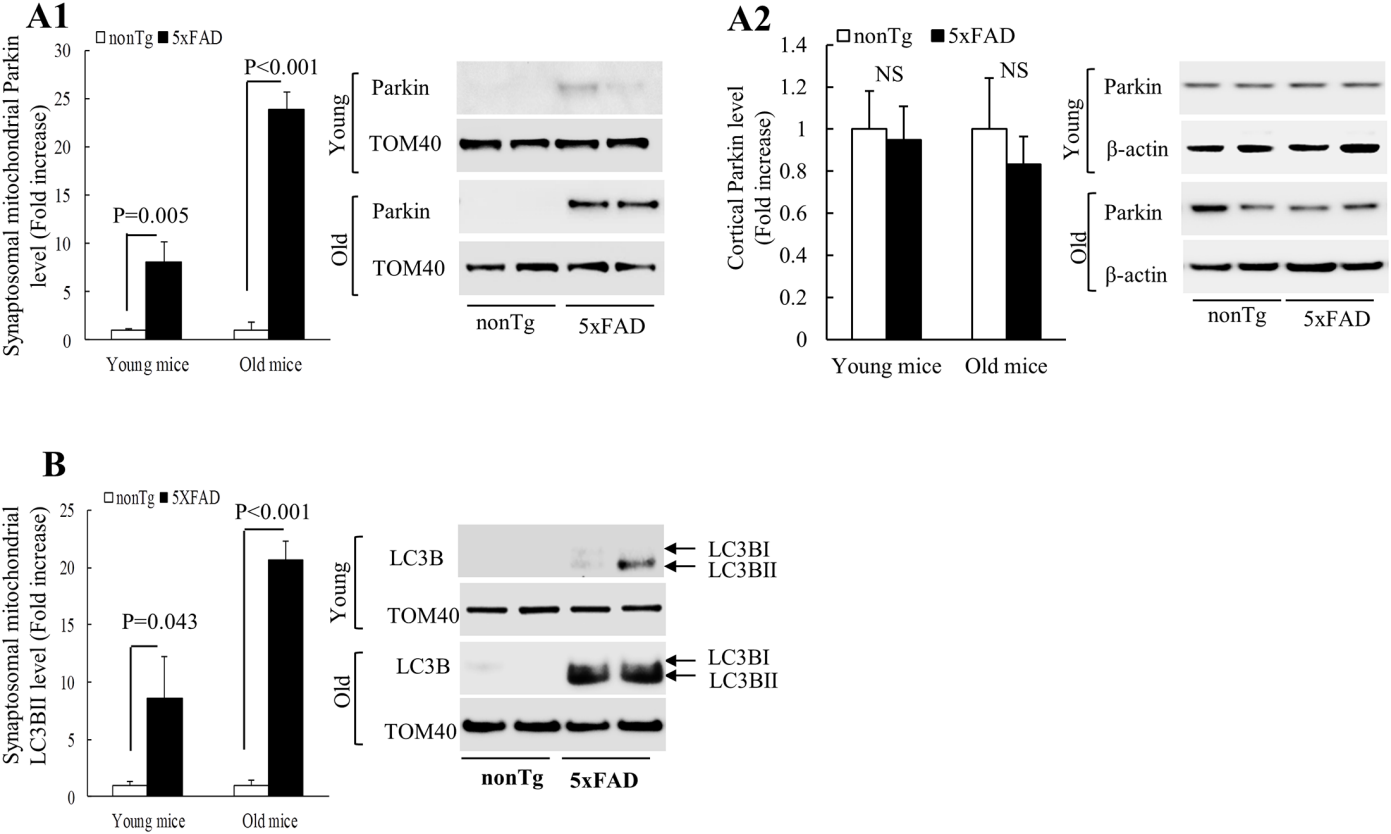
Increased Parkin translocation and LC3BII recruitment in synaptosomal mitochondria from 5xFAD mice. Synaptosomal mitochondria and cortices from young and old nonTg and 5xFAD mice were subjected to the immunoblots for the levels of synaptosomal mitochondrial recruited Parkin (**A1**), the levels of Parkin in cortex (**A2**), and the levels of synaptosomal mitochondria-recruited LC3BII (**B**). The left panels show the analyzed results in fold increase and the right panels are representative immunoblot bands. Mitochondrial TOM40 and β-actin were used as the loading controls for mitochondrial fractions and cortex homogenate, respectively. N = 4–6 mice per group.

To specifically determine whether Parkin translocation to damaged neuronal mitochondria occurs in neurites and at synapses, we employed synaptosomal mitochondria from nonTg and 5xFAD mice at 4 and 9 months old in the study and measured mitochondrial Parkin translocation by immunoblotting. The recruitment of Parkin to mitochondria is the initiative step as well as a strong indicator of Parkin-mediated mitophagy induction [32]. Synaptosomal mitochondria from 5xFAD mice exhibited drastically increased Parkin translocation in an age-dependent manner (Fig 5A1) in comparison to synaptosomal mitochondria from nonTg mice which exhibited little Parkin recruitment. Notably, when we measured the expression level of total Parkin in the cortical extracts, our results showed that there was no significant difference in the total Parkin level in cortices between the two genotypes of mice at the tested ages (Fig 5A2) suggesting that 5xFAD and nonTg mice have similar levels of total Parkin and the increased Parkin translocation to 5xFAD mouse synaptosomal mitochondria is specifically associated with increased mitochondrial impairments.

Increased Parkin translocation to synaptosomal mitochondria is a strong indicator of the activation of Parkin-mediated mitophagy. To determine whether mitophagy is induced in synaptosomal mitochondria in neurites and at synapses from the transgenic mice, we examined the recruitment of LC3BII [33]. Our results showed an age-dependent increase in LC3BII levels in synaptosomal mitochondria from 5xFAD mice in comparison to those from the age-matched nonTg mice (Fig 5B). Indeed, our electromicroscopy (EM) results showed autophagosomes containing mitochondria in the distal ends of neurites in the AD mouse hippocampus (S3 Fig). Therefore, the results seem to implicate that mitophagosome is formed in the distal ends of neurites in the 5xFAD mice in parallel with mitochondrial abnormalities.

### Impaired synaptosomal mitochondrial function correlates to compromised spatial learning & memory of 5xFAD mice

**Fig 6:**
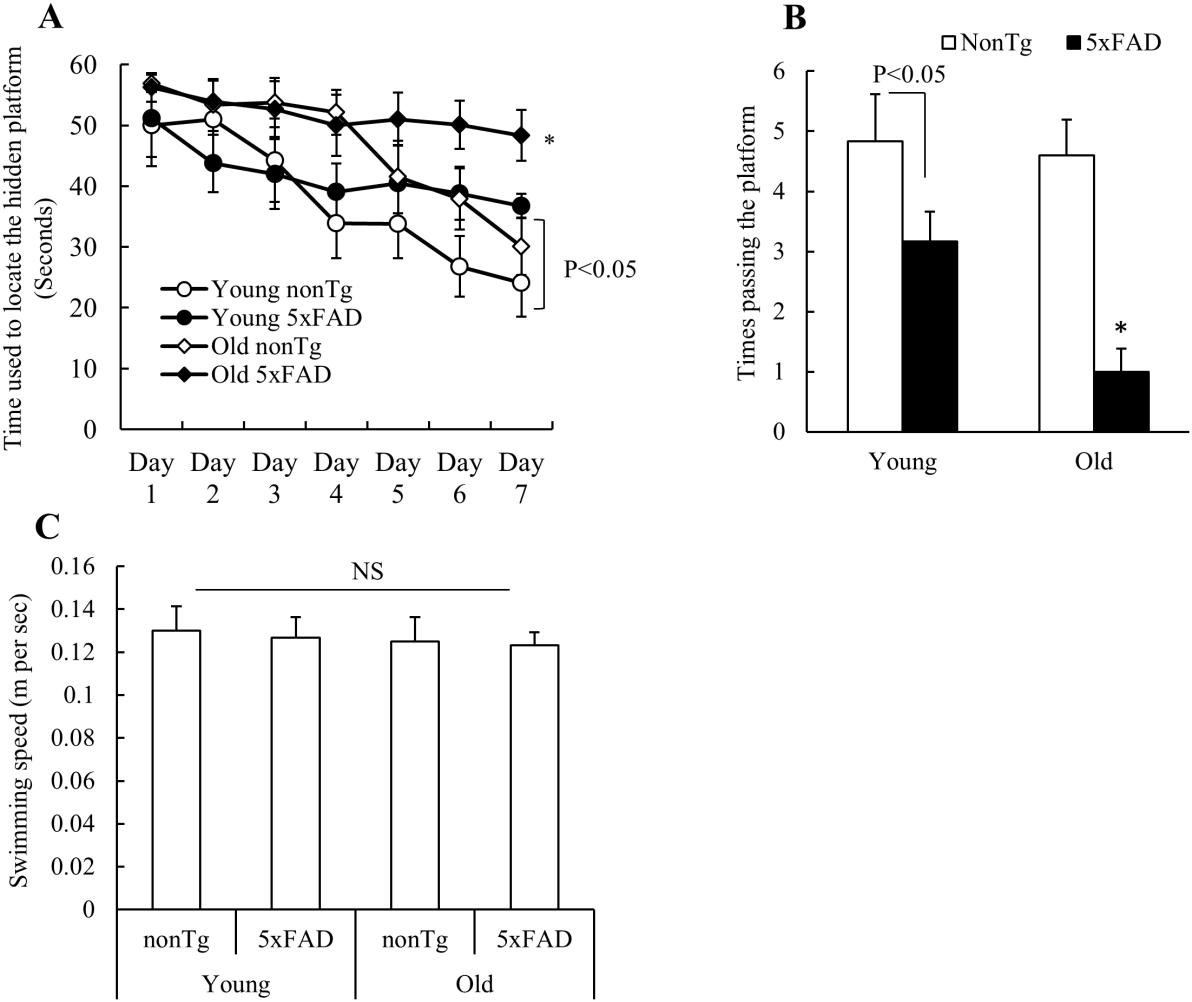
Age-dependent spatial reference learning and memory impairments of 5xFAD mice. 5xFAD mice demonstrated impaired learning ability to locate the hidden platform in an age-dependent manner (**A**). *P<0.05 vs other groups. (**B**) 5xFAD mice had compromised function in spatial reference memory in an age-dependent manner. *P<0.05 vs other groups. (**C**) Mice in different groups didn't show significant change in their swimming speed. N = 5–6 mice of each group.

Compromised spatial learning & memory is the known behavioral alteration in AD and is strongly associated with synaptic injury. In view of the critical role of synaptosomal mitochondria in supporting synaptic function, we examined whether synaptosomal mitochondrial impairments as seen in 5xFAD mice are associated with behavioral changes. 4 and 9 months old 5xFAD and their nonTg littermates were employed to the testing of spatial reference memory by using Morris Water Maze (MWM). 4 months old 5xFAD mice displayed significantly compromised spatial learning & memory in comparison to their age-matched nonTg controls (Fig 6A); whereas the difference in locating the hidden platform between 5xFAD mice and the age-matched nonTg controls was significantly enlarged at the moue age of 9 months (Fig 6A). In the probe test, we removed the platform and measured the times of mouse passing the area where the hidden platform was. 5xFAD mice demonstrated significantly decreased capacity in finding the platform and the decrease was in an age-dependent manner (Fig 6B) with no detectable change in their swimming speed (Fig 6C). Put together, the results suggest the correlation of synaptosomal mitochondrial dysfunctions to compromised spatial learning & memory in 5xFAD mice.

## Discussion

Ever since the recognition of mitochondria as essential organelles in eukaryotic cells, the role of mitochondria in health and disease has been intensively studied. Owing to the progress of mitochondrial biology, mitochondrial heterogeneity has attracted increasing attention in recent years [34]. Synaptosomal mitochondria are a subgroup of neuronal mitochondria and play a critical role in sustaining synaptic functions due to their extreme physical proximity to synapses. In the current study, by using an AD mouse model having the advantages in mimicking human brain amyloidopathy, we have observed early synaptosomal mitochondrial dysfunction in young 5xFAD mice which exacerbates with age and is closely associated with the development of spatial learning & memory decline in this AD mouse model. The results are in agreement to our previous findings by using J20 line [3, 6]. Notably, we have also found that nonsynaptosomal mitochondria from 5xFAD mice displayed significantly lessened and delayed aberrations in comparison to their synaptosomal counterpart. Several factors should be considered to interpret such discrepancy. First, synaptosomal mitochondria are neuron-specific mitochondria; while nonsynaptosomal mitochondria are a mixture of mitochondrial populations from glial cells as well as from the neuronal soma [22]. The greater degree of synaptosomal mitochondrial dysfunction and the relatively preserved nonsynaptosomal mitochondrial function in 5xFAD mice may suggest unparalleled impairments of mitochondrial populations from different types of cells and neuron-specific mitochondria are more vulnerable to Aβ toxicity. In addition, neurons display a typical pattern of mitochondrial heterogeneity and synaptosomal mitochondria differ from their siblings in neuronal soma in many aspects [13, 35–38]. It's generally accepted that neuronal mitochondria are primarily generated in the soma and then delivered to neurites [39, 40]. The relatively longer lifespan, sparse distribution in neurites and heavy workload to support synaptic functions confer susceptibility to synaptosomal mitochondria to accumulate damages. Conceivably, it's not surprising that synaptosomal mitochondria may exhibit more severe injuries than those in the soma in AD-related conditions, which could be supported by our findings that nonsynaptosomal mitochondrial function in 5xFAD mice was comparable to that in nonTg mice at young age when 5xFAD mouse synaptosomal mitochondria exhibited pronounced functional changes. Indeed, to specifically separate synaptosomal and neuronal soma mitochondria from 5xFAD mice will help to clearly address whether synaptosomal mitochondria are more vulnerable to Aβ toxicity than their siblings in the soma. This will be conducted in our future study. Nevertheless, by combining our findings from the two different types of transgenic mouse models expressing human Aβ, our results have ascertained that neuron-specific synaptosomal mitochondrial injury is an early manifestation of brain pathology in Aβ-rich environments which will facilitate our understanding of the role of mitochondrial dysfunction in the development of synaptic injury in AD.

Aβ is thought to be the major mediator of AD [8]. The accumulation of Aβ in mitochondria has been repeatedly identified in AD animal models as well as in AD individuals [3, 5, 20, 21, 41]. Although the detailed impacts of mitochondrial Aβ on mitochondrial function have not been fully depicted yet, it is proposed that the accumulation of Aβ inside mitochondria confers susceptibility to mitochondria in AD-relevant conditions by instigating oxidative stress and compromising the functions of key mitochondrial proteins [3–5, 20, 21, 41]. In this study, we have found early and extensive Aβ accumulation in synaptosomal mitochondria from 5xFAD mice. Accordingly, synaptosomal mitochondria from 5xFAD mice exhibited early and extensive injury in bioenergetics in sharp contrast to their nonsynaptosomal counterpart, which correlates to the patterns of Aβ accumulation. In addition, synaptosomal mitochondria from 5xFAD mice displayed alterations in the balance of mitochondrial fusion and fission proteins and axonal mitochondria from 5xFAD mouse neurons demonstrated a significant elevation of mitochondrial fission indicating that synaptosomal mitochondria from 5xFAD mice undergo deregulated mitochondrial dynamics. Indeed, the aberrations of mitochondrial dynamics have been repeatedly identified in AD brains and AD animal as well as cell models [3, 4, 42, 43]. Compromised mitochondrial bioenergetics induced by Aβ has been suggested to be an involving factor since mitochondrial dynamics and motility are energy consuming processes [3, 4, 42, 43]. Furthermore, a recent study also suggested that the interaction of Aβ with Dlp1 potentiates mitochondrial dynamics alterations [4]. Therefore, our observation of the close association between the progressive accumulation of Aβ in synaptosomal mitochondria and the development of mitochondrial abnormalities in 5xFAD mice supports the deleterious effect of Aβ particularly mitochondrial Aβ on neuronal mitochondrial function.

An intriguing and important finding of this study is the age-dependent elevations of Parkin and LCBII recruitment to synaptosomal mitochondria in 5xFAD mice which correlates to the progressive decline in synaptosomal mitochondrial function. Mitochondrial quality control through mitophagy is a critical pathway to regulate mitochondrial metabolism and maintain mitochondrial fitness [31, 44]. The role of mitophagy in the development of neurodegenerative diseases has received considerable attention in recent years. It is suggested that disrupted mitophagy induction results in blunted mitochondrial turn-over and subsequently leads to the aggregation of defected mitochondria in Parkinson’s disease (PD) patients with mutations in Parkin and/or PINK1 [45, 46]. To the best of our knowledge, conclusive evidence on the state of mitophagy induction in AD, which does not manifest Parkin or PINK dysfunction, has not been obtained yet although increased autophagy has been determined to be featured AD brain pathology [47]. In this study, we have found an age-dependent recruitment of Parkin to synaptosomal mitochondria followed by LC3BII translocation correlating to the progressive development of synaptosomal mitochondrial impairments in 5xFAD mice. The results suggest that mitophagy in synaptosomal mitochondria is activated in the AD mouse model *in vivo*. In addition, we have observed mitophagosomes containing mitochondria in the distal ends of the neurites in 5xFAD mice. The finding has raised an interesting scientific issue of the concurrence of elevated mitophagy induction and damaged synaptosomal mitochondrial accumulation in 5xFAD mice. A simplest interpretation is that elevated mitophagy activation is the cellular response to the fast-developed synaptosomal mitochondrial impairments. However, it cannot be excluded that the mitophagosome clearance mechanisms are also impaired in 5xFAD mice given that efficient removal of damaged mitochondria via mitophagy requires the coordination of two processes including the induction of mitophagy and the clearance of mitophagosomes through lysosome proteolysis [48]. This will form a ground work for our future studies exploring the status of mitophagosome formation and clearance of synaptosomal mitochondria in AD animal models.

Cognitive functions are strongly associated with synaptic activity. Given the crucial role of mitochondria in supporting synaptic function, mitochondrial dysfunction has been linked to synaptic injury and cognitive impairments in AD [49]. In our previous studies, we have found that the protection of mitochondrial function by suppressing mitochondrial permeability transition pore (mPTP) significantly attenuated synaptic injury and cognitive impairments in an AD mouse model [6, 20, 42, 50]. Moreover, recent studies from our group and others conducted on 5xFAD mouse neurons [4, 41, 51, 52] have shown that the application of mitochondria-targeted antioxidants substantially ameliorates neuronal mitochondrial dysfunction and neuronal stress and that altered axonal mitochondrial dynamics are associated with synaptic injury, further supporting the role of mitochondrial abnormalities in inducing neuronal injury and cognitive impairments in AD-relevant pathological settings. It's true that the cognitive decline in AD is a pathological process involving multiple molecular mechanisms including mitochondrial dysfunction. However, in view of the extensive interactions of mitochondria with other molecular mechanisms controlling synaptic plasticity and transmission through mitochondrial role in providing energy and regulating intra-synaptic calcium homeostasis [13–15], mitochondrial dysfunction is at least one mechanism underlying synaptic injury and the resultant cognitive impairments in AD.

In summary, in this study, we have found that neuron-specific synaptosomal mitochondrial population from 5xFAD mice exhibit extensive Aβ accumulation along with the early and progressively exacerbated functional defects as well as elevated Parkin-mediated mitophogy induction. In combination with our previous findings [3, 6], the results have confirmed that synaptosomal mitochondrial dysfunction constitutes an early and primary brain pathology in AD-like Aβ-rich environments, which potentially involves in the development of synaptic injury in AD.

## Supporting Information

S1 Fig The purity of isolated mitochondria. The purity of synaptosomal mitochondria was determined by the abundance of specific mitochondrial proteins (TOM40, COXIV and HSP60) and the absence of calnexin (endoplasmic reticulum) and synaptophysin (synaptic vesicle). (**I**) cortex extracts, (**II**) synaptosomal fractions; (**III**) synaptosomal mitochondrial fractions; and (**IV**) nonsynaptosomal mitochondrial fractions.
(TIF)

S2 Fig The specificity of mitochondrial F1FO ATP synthase β subunit staining. To determine the specificity of mitochondrial F1FO ATP synthase β subunit staining, the antibody was replaced by nonimmue IgG as a critical negative control. Aβ was visualized by the staining of specific antibody. The results showed no nonspecific staining of mitochondrial F1FO ATP synthase β subunit when using the nonimmune IgG to replace its specific antibody. Panels I shows the staining of Aβ (red), NISSL (blue) and Nonimmune IgG (green). Panel II, III and IV represent three-dimension reconstructions of the nonimmune IgG staining, Aβ staining, and merged images, representatively.
(TIF)

S3 Fig Mitophagosome formation in 5xFAD mouse brains. Neurons in hippocampus and neocortex were examined under electromicroscopy for mitophagosome formation. Representative EM image of mitophagosome containing mitochondria in the distal end of a dendrite in 5xFD mice (**A1**). The black arrow indicates the postsynaptic density. (**A2**) is enlarged from the left image. M indicates mitochondria and * indicates mitophagosome containing mitochondria. (**B1**) is representative EM image of mitophagosome at synapses in 5xFAD mice. The black arrow indicates the postsynaptic density. (**B2**) is enlarged image from (**B1**). M indicates mitochondria and * indicates mitophagosome containing mitochondria. (**C1**) is representative EM image of mitophagosome in axons in 5xFAD mice. The black arrow indicates myeline sheath for the determination of axons. (**C2**) and (**C3**) are enlarged images from (**C1**) to show mitophagosomes in axons. (**D**) is a representative image of mitochondria in nonTg mouse hippocampus. N = 3 mice per group.
(TIF)

## References

[1] Johri A, Beal MF. Mitochondrial dysfunction in neurodegenerative diseases. The Journal of pharmacology and experimental therapeutics. 2012;342(3):619–30. 10.1124/jpet.112.192138 22700435 PMC3422529

[2] Lin MT, Beal MF. Mitochondrial dysfunction and oxidative stress in neurodegenerative diseases. Nature. 2006;443(7113):787–95. 10.1038/nature05292 . 17051205

[3] Du H, Guo L, Yan S, Sosunov AA, McKhann GM, Yan SS. Early deficits in synaptic mitochondria in an Alzheimer's disease mouse model. Proceedings of the National Academy of Sciences of the United States of America. 2010;107(43):18670–5. 10.1073/pnas.1006586107 20937894 PMC2972922

[4] Calkins MJ, Reddy PH. Amyloid beta impairs mitochondrial anterograde transport and degenerates synapses in Alzheimer's disease neurons. Biochimica et biophysica acta. 2011;1812(4):507–13. 10.1016/j.bbadis.2011.01.007 21241801 PMC3042500

[5] Lustbader JW, Cirilli M, Lin C, Xu HW, Takuma K, Wang N, et al ABAD directly links Abeta to mitochondrial toxicity in Alzheimer's disease. Science. 2004;304(5669):448–52. 10.1126/science.1091230 . 15087549

[6] Du H, Guo L, Wu X, Sosunov AA, McKhann GM, Chen JX, et al Cyclophilin D deficiency rescues Abeta-impaired PKA/CREB signaling and alleviates synaptic degeneration. Biochimica et biophysica acta. 2014;1842(12 Pt A):2517–27. 10.1016/j.bbadis.2013.03.004 23507145 PMC3868643

[7] Yao J, Irwin RW, Zhao L, Nilsen J, Hamilton RT, Brinton RD. Mitochondrial bioenergetic deficit precedes Alzheimer's pathology in female mouse model of Alzheimer's disease. Proceedings of the National Academy of Sciences of the United States of America. 2009;106(34):14670–5. 10.1073/pnas.0903563106 19667196 PMC2732886

[8] Reddy PH, Beal MF. Amyloid beta, mitochondrial dysfunction and synaptic damage: implications for cognitive decline in aging and Alzheimer's disease. Trends in molecular medicine. 2008;14(2):45–53. 10.1016/j.molmed.2007.12.002 18218341 PMC3107703

[9] Reddy PH. Mitochondrial dysfunction in aging and Alzheimer's disease: strategies to protect neurons. Antioxidants & redox signaling. 2007;9(10):1647–58. 10.1089/ars.2007.1754 . 17696767

[10] Du H, Guo L, Yan SS. Synaptic mitochondrial pathology in Alzheimer's disease. Antioxidants & redox signaling. 2012;16(12):1467–75. 10.1089/ars.2011.4277 21942330 PMC3329948

[11] Reddy PH, Manczak M, Mao P, Calkins MJ, Reddy AP, Shirendeb U. Amyloid-beta and mitochondria in aging and Alzheimer's disease: implications for synaptic damage and cognitive decline. Journal of Alzheimer's disease: JAD. 2010;20 Suppl 2:S499–512. 10.3233/JAD-2010-100504 20413847 PMC3059092

[12] Mungarro-Menchaca X, Ferrera P, Moran J, Arias C. beta-Amyloid peptide induces ultrastructural changes in synaptosomes and potentiates mitochondrial dysfunction in the presence of ryanodine. Journal of neuroscience research. 2002;68(1):89–96. . 11933053 10.1002/jnr.10193

[13] Ly CV, Verstreken P. Mitochondria at the synapse. The Neuroscientist: a review journal bringing neurobiology, neurology and psychiatry. 2006;12(4):291–9. 10.1177/1073858406287661 . 16840705

[14] Banaclocha MM, Hernandez AI, Martinez N, Ferrandiz ML. N-acetylcysteine protects against age-related increase in oxidized proteins in mouse synaptic mitochondria. Brain research. 1997;762(1–2):256–8. . 9262186 10.1016/s0006-8993(97)00493-9

[15] Martinez M, Hernandez AI, Martinez N, Ferrandiz ML. Age-related increase in oxidized proteins in mouse synaptic mitochondria. Brain research. 1996;731(1–2):246–8. . 8883880 10.1016/0006-8993(96)00708-1

[16] Lee JE, Han PL. An update of animal models of Alzheimer disease with a reevaluation of plaque depositions. Experimental neurobiology. 2013;22(2):84–95. 10.5607/en.2013.22.2.84 23833557 PMC3699678

[17] Maarouf CL, Kokjohn TA, Whiteside CM, Macias MP, Kalback WM, Sabbagh MN, et al Molecular Differences and Similarities Between Alzheimer's Disease and the 5XFAD Transgenic Mouse Model of Amyloidosis. Biochemistry insights. 2013;6:1–10. 10.4137/BCI.S13025 25210460 PMC4154482

[18] Eimer WA, Vassar R. Neuron loss in the 5XFAD mouse model of Alzheimer's disease correlates with intraneuronal Abeta42 accumulation and Caspase-3 activation. Molecular neurodegeneration. 2013;8:2 10.1186/1750-1326-8-2 23316765 PMC3552866

[19] Oakley H, Cole SL, Logan S, Maus E, Shao P, Craft J, et al Intraneuronal beta-amyloid aggregates, neurodegeneration, and neuron loss in transgenic mice with five familial Alzheimer's disease mutations: potential factors in amyloid plaque formation. The Journal of neuroscience: the official journal of the Society for Neuroscience. 2006;26(40):10129–40. 10.1523/JNEUROSCI.1202-06.2006 . 17021169 PMC6674618

[20] Du H, Guo L, Fang F, Chen D, Sosunov AA, McKhann GM, et al Cyclophilin D deficiency attenuates mitochondrial and neuronal perturbation and ameliorates learning and memory in Alzheimer's disease. Nature medicine. 2008;14(10):1097–105. 10.1038/nm.1868 18806802 PMC2789841

[21] Fang D, Wang Y, Zhang Z, Du H, Yan S, Sun Q, et al Increased neuronal PreP activity reduces Abeta accumulation, attenuates neuroinflammation and improves mitochondrial and synaptic function in Alzheimer disease's mouse model. Human molecular genetics. 2015;24(18):5198–210. Epub 2015/07/01. 10.1093/hmg/ddv241 26123488 PMC4550821

[22] Dunkley PR, Jarvie PE, Robinson PJ. A rapid Percoll gradient procedure for preparation of synaptosomes. Nature protocols. 2008;3(11):1718–28. 10.1038/nprot.2008.171 . 18927557

[23] Cavallucci V, Bisicchia E, Cencioni MT, Ferri A, Latini L, Nobili A, et al Acute focal brain damage alters mitochondrial dynamics and autophagy in axotomized neurons. Cell death & disease. 2014;5:e1545 10.1038/cddis.2014.511 25429622 PMC4260762

[24] Frank M, Duvezin-Caubet S, Koob S, Occhipinti A, Jagasia R, Petcherski A, et al Mitophagy is triggered by mild oxidative stress in a mitochondrial fission dependent manner. Biochimica et biophysica acta. 2012;1823(12):2297–310. 10.1016/j.bbamcr.2012.08.007 . 22917578

[25] Medana IM, Esiri MM. Axonal damage: a key predictor of outcome in human CNS diseases. Brain: a journal of neurology. 2003;126(Pt 3):515–30. . 12566274 10.1093/brain/awg061

[26] Cai Q, Zakaria HM, Simone A, Sheng ZH. Spatial parkin translocation and degradation of damaged mitochondria via mitophagy in live cortical neurons. Current biology: CB. 2012;22(6):545–52. 10.1016/j.cub.2012.02.005 22342752 PMC3313683

[27] Bin-Umer MA, McLaughlin JE, Butterly MS, McCormick S, Tumer NE. Elimination of damaged mitochondria through mitophagy reduces mitochondrial oxidative stress and increases tolerance to trichothecenes. Proceedings of the National Academy of Sciences of the United States of America. 2014;111(32):11798–803. 10.1073/pnas.1403145111 25071194 PMC4136610

[28] Matsuda N, Sato S, Shiba K, Okatsu K, Saisho K, Gautier CA, et al PINK1 stabilized by mitochondrial depolarization recruits Parkin to damaged mitochondria and activates latent Parkin for mitophagy. The Journal of cell biology. 2010;189(2):211–21. 10.1083/jcb.200910140 20404107 PMC2856912

[29] Seibler P, Graziotto J, Jeong H, Simunovic F, Klein C, Krainc D. Mitochondrial Parkin recruitment is impaired in neurons derived from mutant PINK1 induced pluripotent stem cells. The Journal of neuroscience: the official journal of the Society for Neuroscience. 2011;31(16):5970–6. 10.1523/JNEUROSCI.4441-10.2011 21508222 PMC3091622

[30] Van Laar VS, Arnold B, Cassady SJ, Chu CT, Burton EA, Berman SB. Bioenergetics of neurons inhibit the translocation response of Parkin following rapid mitochondrial depolarization. Human molecular genetics. 2011;20(5):927–40. 10.1093/hmg/ddq531 21147754 PMC3033183

[31] Ashrafi G, Schlehe JS, LaVoie MJ, Schwarz TL. Mitophagy of damaged mitochondria occurs locally in distal neuronal axons and requires PINK1 and Parkin. The Journal of cell biology. 2014;206(5):655–70. 10.1083/jcb.201401070 25154397 PMC4151150

[32] Vives-Bauza C, Zhou C, Huang Y, Cui M, de Vries RL, Kim J, et al PINK1-dependent recruitment of Parkin to mitochondria in mitophagy. Proceedings of the National Academy of Sciences of the United States of America. 2010;107(1):378–83. 10.1073/pnas.0911187107 19966284 PMC2806779

[33] Narendra D, Kane LA, Hauser DN, Fearnley IM, Youle RJ. p62/SQSTM1 is required for Parkin-induced mitochondrial clustering but not mitophagy; VDAC1 is dispensable for both. Autophagy. 2010;6(8):1090–106. 20890124 10.4161/auto.6.8.13426 PMC3359490

[34] Kuznetsov AV, Margreiter R. Heterogeneity of mitochondria and mitochondrial function within cells as another level of mitochondrial complexity. International journal of molecular sciences. 2009;10(4):1911–29. 10.3390/ijms10041911 19468346 PMC2680654

[35] Zenisek D, Matthews G. The role of mitochondria in presynaptic calcium handling at a ribbon synapse. Neuron. 2000;25(1):229–37. . 10707986 10.1016/s0896-6273(00)80885-5

[36] Hollenbeck PJ. Mitochondria and neurotransmission: evacuating the synapse. Neuron. 2005;47(3):331–3. 10.1016/j.neuron.2005.07.017 16055057 PMC2538582

[37] Reddy PH, Tripathi R, Troung Q, Tirumala K, Reddy TP, Anekonda V, et al Abnormal mitochondrial dynamics and synaptic degeneration as early events in Alzheimer's disease: implications to mitochondria-targeted antioxidant therapeutics. Biochimica et biophysica acta. 2012;1822(5):639–49. 10.1016/j.bbadis.2011.10.011 22037588 PMC3272314

[38] Calkins MJ, Manczak M, Mao P, Shirendeb U, Reddy PH. Impaired mitochondrial biogenesis, defective axonal transport of mitochondria, abnormal mitochondrial dynamics and synaptic degeneration in a mouse model of Alzheimer's disease. Human molecular genetics. 2011;20(23):4515–29. 10.1093/hmg/ddr381 21873260 PMC3209824

[39] Chang DT, Reynolds IJ. Differences in mitochondrial movement and morphology in young and mature primary cortical neurons in culture. Neuroscience. 2006;141(2):727–36. 10.1016/j.neuroscience.2006.01.034 . 16797853

[40] Glater EE, Megeath LJ, Stowers RS, Schwarz TL. Axonal transport of mitochondria requires milton to recruit kinesin heavy chain and is light chain independent. The Journal of cell biology. 2006;173(4):545–57. 10.1083/jcb.200601067 16717129 PMC2063864

[41] Manczak M, Anekonda TS, Henson E, Park BS, Quinn J, Reddy PH. Mitochondria are a direct site of A beta accumulation in Alzheimer's disease neurons: implications for free radical generation and oxidative damage in disease progression. Human molecular genetics. 2006;15(9):1437–49. 10.1093/hmg/ddl066 . 16551656

[42] Guo L, Du H, Yan S, Wu X, McKhann GM, Chen JX, et al Cyclophilin D deficiency rescues axonal mitochondrial transport in Alzheimer's neurons. PloS one. 2013;8(1):e54914 10.1371/journal.pone.0054914 23382999 PMC3561411

[43] Zhu X, Perry G, Smith MA, Wang X. Abnormal mitochondrial dynamics in the pathogenesis of Alzheimer's disease. Journal of Alzheimer's disease: JAD. 2013;33 Suppl 1:S253–62. 10.3233/JAD-2012-129005 22531428 PMC4097015

[44] Ashrafi G, Schwarz TL. PINK1- and PARK2-mediated local mitophagy in distal neuronal axons. Autophagy. 2015:11 . 25607607 10.1080/15548627.2014.996021 PMC4502792

[45] Vives-Bauza C, Przedborski S. Mitophagy: the latest problem for Parkinson's disease. Trends in molecular medicine. 2011;17(3):158–65. 10.1016/j.molmed.2010.11.002 . 21146459

[46] Chu CT. Diversity in the regulation of autophagy and mitophagy: lessons from Parkinson's disease. Parkinson's disease. 2011;2011:789431 10.4061/2011/789431 21603187 PMC3096099

[47] Boland B, Kumar A, Lee S, Platt FM, Wegiel J, Yu WH, et al Autophagy induction and autophagosome clearance in neurons: relationship to autophagic pathology in Alzheimer's disease. The Journal of neuroscience: the official journal of the Society for Neuroscience. 2008;28(27):6926–37. 10.1523/JNEUROSCI.0800-08.2008 18596167 PMC2676733

[48] Hattori N, Saiki S, Imai Y. Regulation by mitophagy. The international journal of biochemistry & cell biology. 2014;53:147–50. 10.1016/j.biocel.2014.05.012 . 24842103

[49] Reddy PH, Beal MF. Are mitochondria critical in the pathogenesis of Alzheimer's disease? Brain research Brain research reviews. 2005;49(3):618–32. 10.1016/j.brainresrev.2005.03.004 . 16269322

[50] Du H, Guo L, Zhang W, Rydzewska M, Yan S. Cyclophilin D deficiency improves mitochondrial function and learning/memory in aging Alzheimer disease mouse model. Neurobiology of aging. 2011;32(3):398–406. 10.1016/j.neurobiolaging.2009.03.003 19362755 PMC3304024

[51] Manczak M, Mao P, Calkins MJ, Cornea A, Reddy AP, Murphy MP, et al Mitochondria-targeted antioxidants protect against amyloid-beta toxicity in Alzheimer's disease neurons. Journal of Alzheimer's disease: JAD. 2010;20 Suppl 2:S609–31. 10.3233/JAD-2010-100564 20463406 PMC3072711

[52] Lu L, Guo L, Gauba E, Tian J, Wang L, Tandon N, et al Transient Cerebral Ischemia Promotes Brain Mitochondrial Dysfunction and Exacerbates Cognitive Impairments in Young 5xFAD Mice. PloS one. 2015;10(12):e0144068 10.1371/journal.pone.0144068 26632816 PMC4669173

[53] Yang DS, Lee JH, Nixon RA. Monitoring autophagy in Alzheimer's disease and related neurodegenerative diseases. Methods in enzymology. 2009;453:111–44. 10.1016/S0076-6879(08)04006-8 . 19216904

[54] Vorhees CV, Williams MT. Morris water maze: procedures for assessing spatial and related forms of learning and memory. Nat Protocols. 2006;1(2):848–58. 17406317 10.1038/nprot.2006.116 PMC2895266

