# Supplementary figures and images for: Synaptosomal Mitochondrial Dysfunction in 5xFAD Mouse Model of Alzheimer's Disease
Source: PLoS One. 2016 Mar 4;11(3):e0150441. doi: 10.1371/journal.pone.0150441 (PMC4778903; doi:10.1371/journal.pone.0150441)

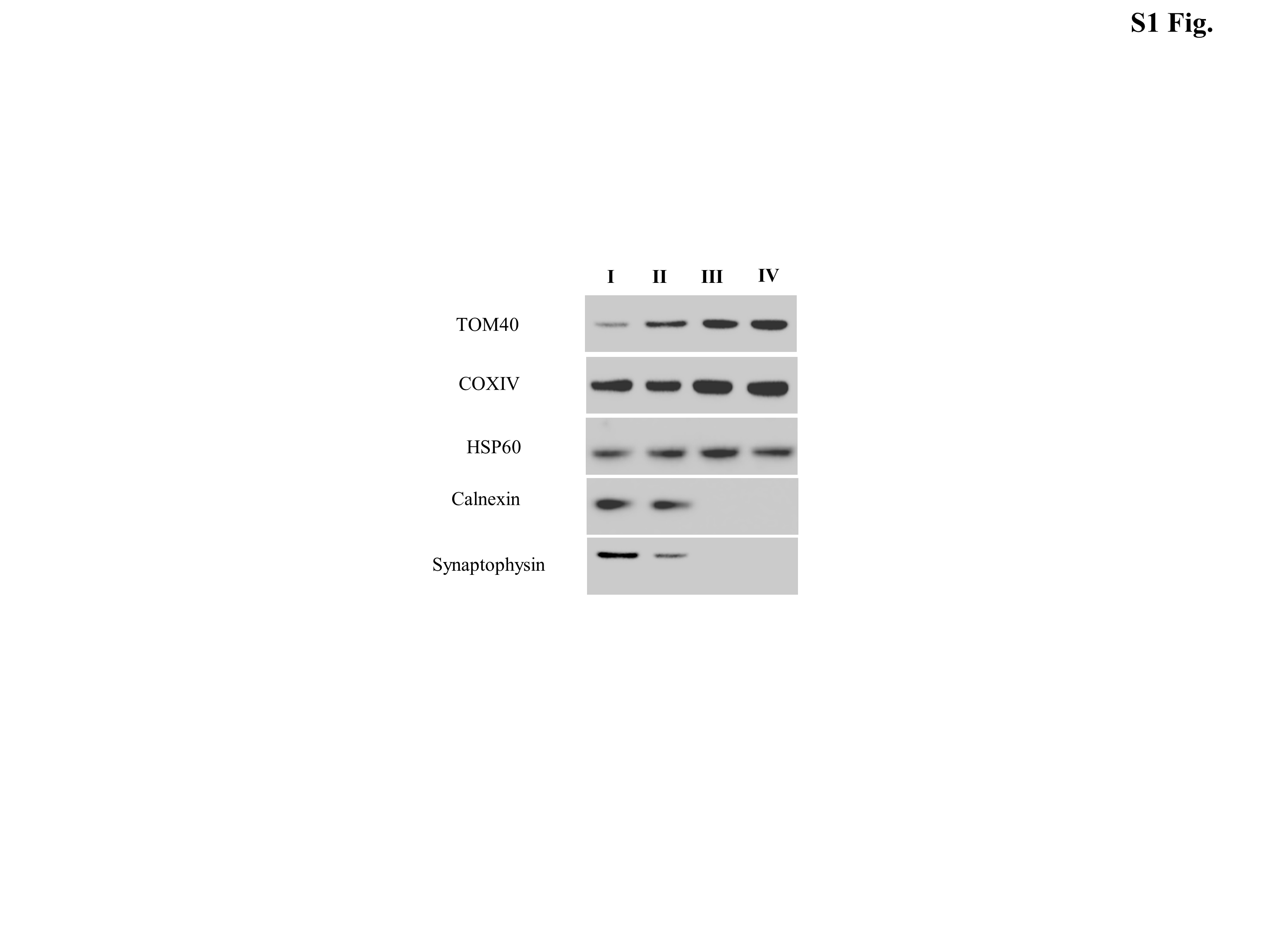

Supplement: S1 Fig — The purity of synaptosomal mitochondria was determined by the abundance of specific mitochondrial proteins (TOM40, COXIV and HSP60) and the absence of calnexin (endoplasmic reticulum) and synaptophysin (synaptic vesicle). (I) cortex extracts, (II) synaptosomal fractions; (III) synaptosomal mitochondrial fractions; and (IV) nonsynaptosomal mitochondrial fractions. (TIF) [file pone.0150441.s001.tif]

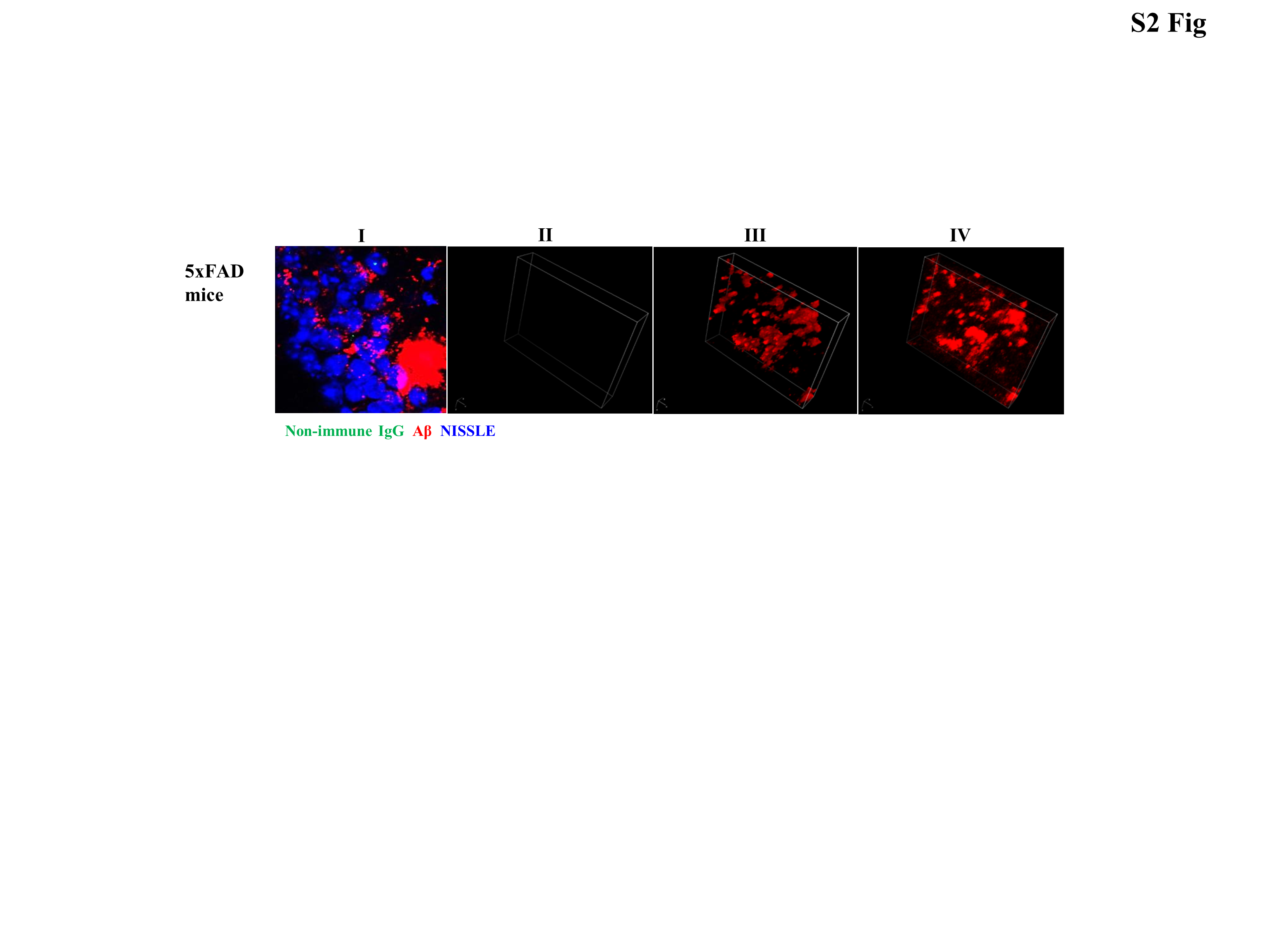

Supplement: S2 Fig — To determine the specificity of mitochondrial F1FO ATP synthase β subunit staining, the antibody was replaced by nonimmue IgG as a critical negative control. Aβ was visualized by the staining of specific antibody. The results showed no nonspecific staining of mitochondrial F1FO ATP synthase β subunit when using the nonimmune IgG to replace its specific antibody. Panels I shows the staining of Aβ (red), NISSL (blue) and Nonimmune IgG (green). Panel II, III and IV represent three-dimension reconstructions of the nonimmune IgG staining, Aβ staining, and merged images, representatively. (TIF) [file pone.0150441.s002.tif]

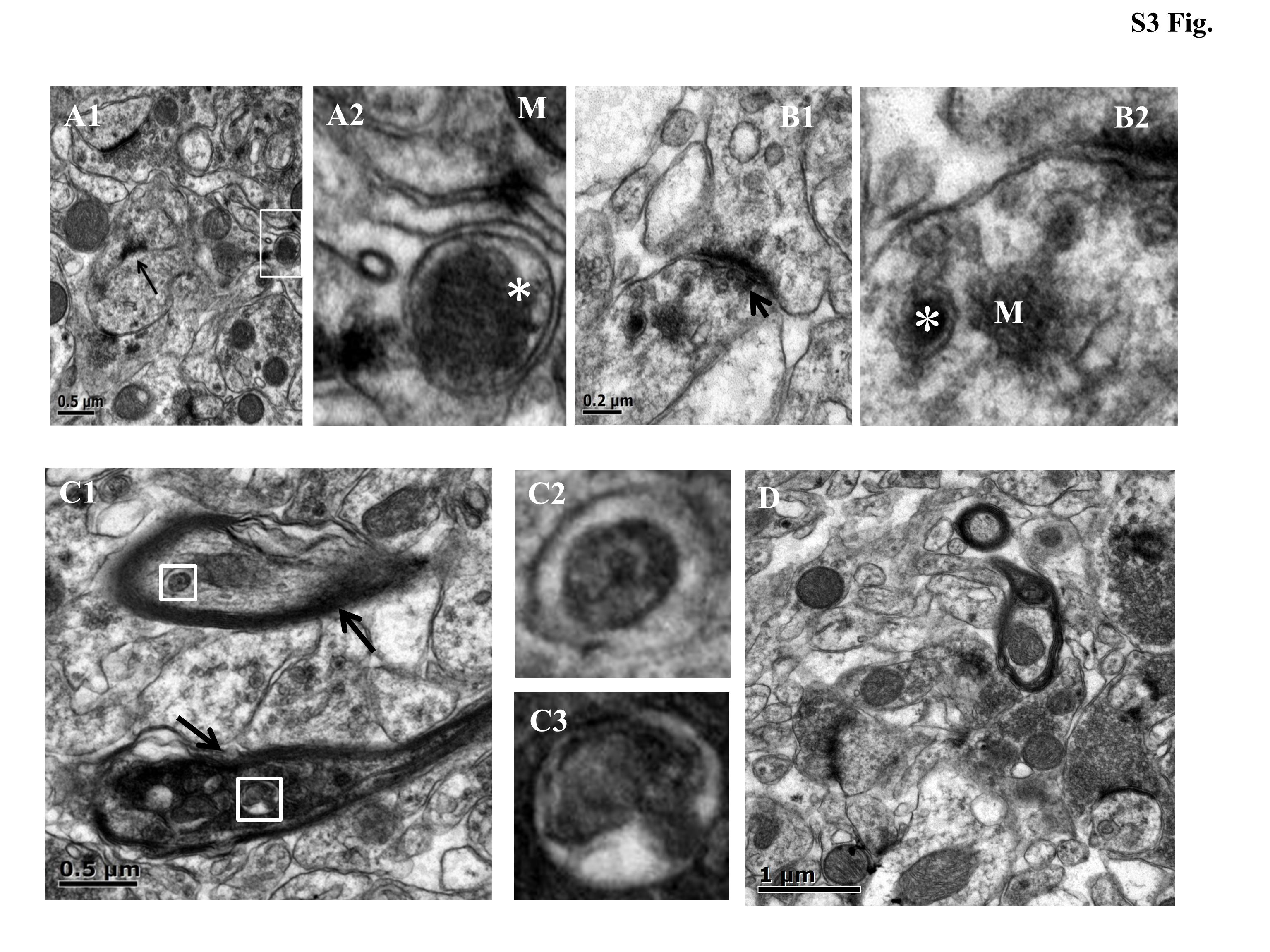

Supplement: S3 Fig — Neurons in hippocampus and neocortex were examined under electromicroscopy for mitophagosome formation. Representative EM image of mitophagosome containing mitochondria in the distal end of a dendrite in 5xFD mice (A1). The black arrow indicates the postsynaptic density. (A2) is enlarged from the left image. M indicates mitochondria and * indicates mitophagosome containing mitochondria. (B1) is representative EM image of mitophagosome at synapses in 5xFAD mice. The black arrow indicates the postsynaptic density. (B2) is enlarged image from (B1). M indicates mitochondria and * indicates mitophagosome containing mitochondria. (C1) is representative EM image of mitophagosome in axons in 5xFAD mice. The black arrow indicates myeline sheath for the determination of axons. (C2) and (C3) are enlarged images from (C1) to show mitophagosomes in axons. (D) is a representative image of mitochondria in nonTg mouse hippocampus. N = 3 mice per group. (TIF) [file pone.0150441.s003.tif]
